# Protective Effect of Tyrosol on BALF Cytology and Biochemistry in Rats Administered Intratracheal Bleomycin

**DOI:** 10.3390/vetsci12080760

**Published:** 2025-08-14

**Authors:** Elif Ekinci, Burak Karabulut, Canan Akdeniz Incili, Eren Cankaya, Ibrahim Seker, Necati Timurkaan

**Affiliations:** 1 Department of Pathology, Faculty of Veterinary Medicine, University of Dicle, Diyarbakir 21200, Türkiye; 2 Department of Pathology, Faculty of Veterinary Medicine, University of Firat, Elazig 23200, Türkiye; bkarabulut@firat.edu.tr (B.K.); caincili@firat.edu.tr (C.A.I.); ecankaya@firat.edu.tr (E.C.); ntimurkaan@firat.edu.tr (N.T.); 3 Department of Zootechnics, Faculty of Veterinary Medicine, University of Firat, Elazig 23200, Türkiye; iseker@firat.edu.tr

**Keywords:** bleomycin, BALF, foamy macrophage

## Abstract

This study aimed to investigate the cytological and biochemical effects of tyrosol on bronchoalveolar lavage fluid (BALF) in an experimental lung injury model induced via intratracheal bleomycin (BLM) administration at 4 mg/kg. Tyrosol is a compound found in olive oil with antioxidant, anti-inflammatory, and antifibrotic activity, and there are no publications on its effect on broncho-alveolar lavage. A total of fifty male Sprague Dawley rats were randomly divided into five groups: control, BLM only, and BLM combined with tyrosol at doses of 20, 40, and 80 mg/kg. Following a two-week treatment period, BALF samples were collected and evaluated cytologically and biochemically. BLM administration led to significant increases in the proportions of lymphocytes, neutrophils, and epithelial cells (*p* < 0.05) and a decrease in macrophage percentages in BALF. Tyrosol treatment modulated these cellular alterations in a dose-dependent manner, with notable increases in macrophage ratios and reductions in inflammatory cells, particularly at 40 and 80 mg/kg doses. Furthermore, the presence of foamy macrophages—commonly observed in the BLM group—was found to decrease in a dose-dependent manner with tyrosol administration. Biochemical analyses showed that BLM significantly elevated malondialdehyde (MDA) levels (*p* < 0.05), while reducing the levels of antioxidant enzymes such as superoxide dismutase (SOD), glutathione peroxidase (GPx), and catalase (CAT). Tyrosol treatment improved these parameters in a dose-dependent manner, thereby reducing oxidative stress. In cytokine analysis, BLM increased all proinflammatory cytokine levels, whereas tyrosol treatment, particularly at higher doses, significantly decreased IL-6 levels (*p* < 0.05). In conclusion, tyrosol demonstrated notable protective effects against bleomycin-induced lung injury by exerting anti-inflammatory and antioxidative actions at the BALF level.

## Simple Summary

This study evaluated the effects of tyrosol, a natural compound found in olive oil, in a bleomycin (BLM)-induced experimental lung injury model in rats. Following the intratracheal administration of 4 mg/kg BLM, rats received tyrosol at doses of 20, 40, or 80 mg/kg. After two weeks, bronchoalveolar lavage fluid (BALF) was analyzed. BLM caused increased inflammation and decreased macrophage ratios, while tyrosol treatment reversed these changes in a dose-dependent manner. Additionally, foamy macrophages, MDA levels, and IL-6 were significantly reduced by tyrosol. These findings suggest that tyrosol exerts anti-inflammatory and antioxidant effects, providing protection against BLM-induced lung injury.

## 1. Introduction

Although clinical findings, laboratory tests, pulmonary function analysis, imaging techniques, and biopsies remain essential tools in the diagnosis of pulmonary diseases, these conventional methods can sometimes be inadequate or limited in use due to their invasive nature [1]. At this point, bronchoalveolar lavage (BAL) stands out as a minimally invasive and valuable technique, particularly in the differential diagnosis of interstitial and diffuse lung diseases [2]. Bronchoalveolar lavage fluid (BALF), obtained via BAL, provides direct information on alveolar-level cellular responses, the degree of inflammation, and biochemical content. Thus, BALF contributes significantly to both diagnostic and therapeutic research. Particularly, in experimental models, BALF analysis has become a critical tool in assessing the extent of lung injury through cellular composition, cytokine profiles, and oxidative stress parameters [3]. Bleomycin (BLM), an antibiotic isolated from Streptomyces verticillus, is widely used in the treatment of lymphomas and testicular germ cell tumors [4]. However, due to its side effect of inducing pulmonary fibrosis, BLM is commonly employed to model experimental pulmonary fibrosis in animal studies [5]. The bleomycin-induced lung injury model is frequently used to evaluate inflammatory and fibrotic responses [6]. BLM has been reported to cause increases in proinflammatory cytokines and marked cellular infiltration in lung tissue, leading to significant alterations in BALF parameters [3,7,8]. In recent years, the potential therapeutic effects of naturally occurring phenolic compounds against such injuries have been investigated. One such compound, tyrosol, has attracted attention for its antioxidant [9] and anti-inflammatory [10] properties. However, its effects on the lungs, particularly its influence on BALF in bleomycin-induced injury models, have not been thoroughly elucidated. This study aims to investigate the cytological and biochemical effects of tyrosol on BALF in a model of lung injury induced via intratracheal bleomycin administration.

## 2. Materials and Methods

### 2.1. Animal Supply and Care Conditions and Chemicals

A total of 50 male Sprague Dawley (SD) rats, aged 8–12 weeks, were obtained from the Fırat University Experimental Research Center (FUDAM). The study was approved by the Fırat University Local Ethics Committee for Animal Experiments with the protocol number 315276, dated 18 April 2023. All procedures involving animals were carried out in accordance with the guidelines set by the Fırat University Local Ethics Committee for Animal Experiments. To ensure acclimatization, animals were subjected to a one-week adaptation period before the start of the experiment. Animal care was provided at the Fırat University Experimental Research Center (FUDAM). All rats were fed standard rodent chow and tap water ad libitum. They were housed in plastic cages with stainless steel wire lids, kept at room temperature (22 ± 2 °C), under a 12 h light/dark cycle. The chemicals used in this study, such as bleomycin sulfate (Blemisin lyophilized powder, Koçak Farma, Tekirdağ, Türkiye) and tyrosol (Tokyo Chemical Industry Co., Ltd.,6-15-9 Toshima, Kita-Ku, Tokyo, Japan), were supplied by commercial companies. The bleomycin dose was determined based on previous studies [11,12]. The tyrosol doses were selected according to the doses safely used in earlier studies [13,14].

### 2.2. Animal Group

In this study, a total of fifty Sprague Dawley rats were randomly divided into five groups of ten animals each.

Group 1 (SS + distilled water) served as the control group; animals received 0.25 mL of sterile physiological saline intratracheally (i.t.) and 1 mL/day of distilled water via gastric gavage for 14 days.

Group 2 (BLM + distilled water) served as the disease model group; animals received 4 mg/kg of bleomycin (BLM) dissolved in 0.25 mL of saline intratracheally, followed by 1 mL/day of distilled water for 14 days.

Groups 3, 4, and 5 (BLM + Tyrosol 20, 40, 80 mg/kg) received the same BLM administration as in Group 2. Additionally, tyrosol was administered at doses of 20, 40, and 80 mg/kg, respectively, dissolved in distilled water, and given via gastric gavage for 14 consecutive days.

### 2.3. Experimental Procedures

Day 1 of the experiment was defined as the day of the intratracheal administration of either bleomycin or saline. All intratracheal procedures were performed under general anesthesia. Anesthesia was induced via intraperitoneal injection of 5 mg/kg xylazine and 50 mg/kg ketamine. Adequate anesthetic depth was confirmed via the absence of reflexes.

**Figure 1:**
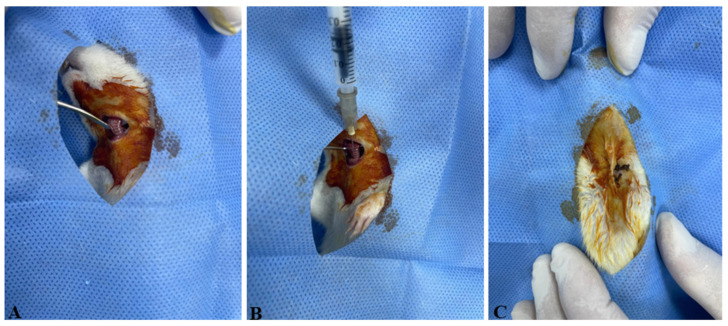
The experimental procedures performed on the animals. (**A**) The trachea was exposed by carefully retracting the subcutaneous tissues, adipose tissue, connective tissue, and vascular structures with the aid of a clamp. (**B**) A 1 mL insulin syringe was inserted into the trachea to administer sterile saline (SS) to Group 1 and bleomycin (BLM) to Groups 2, 3, 4, and 5. (**C**) The incision site was sutured using 4/0 atraumatic silk suture with a sharp-bodied needle.

Following the induction of anesthesia, a midline cervical incision was made to expose the trachea (Figure 1A). The subcutaneous tissue, adipose tissue, connective tissue, and surrounding vascular structures were gently retracted using a clamp to visualize the tracheal lumen. A 1 mL insulin syringe was used to deliver the test solutions directly into the trachea (Figure 1B). Group 1 received 0.25 mL of sterile saline, while Groups 2 to 5 received a single intratracheal dose of 4 mg/kg bleomycin dissolved in saline. To ensure effective pulmonary delivery of the solution, the rats were held at a 45-degree incline with their heads elevated for 30 s. The surgical incision was then closed using 4/0 atraumatic silk sutures (Figure 1C), and daily postoperative wound care was provided for one week.

### 2.4. Collection and Processing of Bronchoalveolar Lavage Fluid (BALF)

Following euthanasia via intracardiac blood collection under anesthesia, the thoracic cavity was opened, and bronchoalveolar lavage fluid (BALF) was collected by flushing the lungs three times with 3 mL of sterile saline via an intratracheal cannula. The collected BALF from each rat was centrifuged at 4000 rpm for 10 min. The resulting supernatant was stored at −80 °C for subsequent cytokine analysis. For cytological examination, phosphate-buffered saline (PBS) was added to the cell pellet and centrifuged again at 1200× *g* for 10 min. In the evaluation of BALF samples, the method reported by Poitout-Belissent et al. (2021) was modified and applied [15]. Following the removal of the supernatant, the remaining pellet was spread onto glass slides, fixed with alcohol, and stained with Giemsa. For each sample, 200 nucleated cells were counted, and cell types were evaluated in terms of their absolute numbers and their proportions within the total cell population.

### 2.5. Biochemical Analysis of BALF

The levels of MDA, CAT, SOD, GPx, IL-1β, IL-6, IL-13, TNF-α, and TGF-β1 in the BALF were quantitatively measured using enzyme-linked immunosorbent assay (ELISA) kits (Table 1).

### 2.6. Statistical Analysis

Descriptive statistics were initially calculated for all evaluated parameters. The data were assessed for compliance with the assumptions of parametric tests. For comparisons among groups, one-way analysis of variance (ANOVA) was used, and Duncan’s multiple range test was applied for post hoc analysis when significant differences were detected. For parameters not meeting parametric assumptions, the Kruskal–Wallis test was applied, followed by the Bonferroni-adjusted Mann–Whitney U test for pairwise comparisons [16]. A significance level of *p* < 0.05 was accepted for all statistical tests. Statistical analyses were performed using SPSS software version 22.0 (SPSS Inc., Chicago, IL, USA, 2015).

## 3. Results

### 3.1. Cytological Results

**Figure 2:**
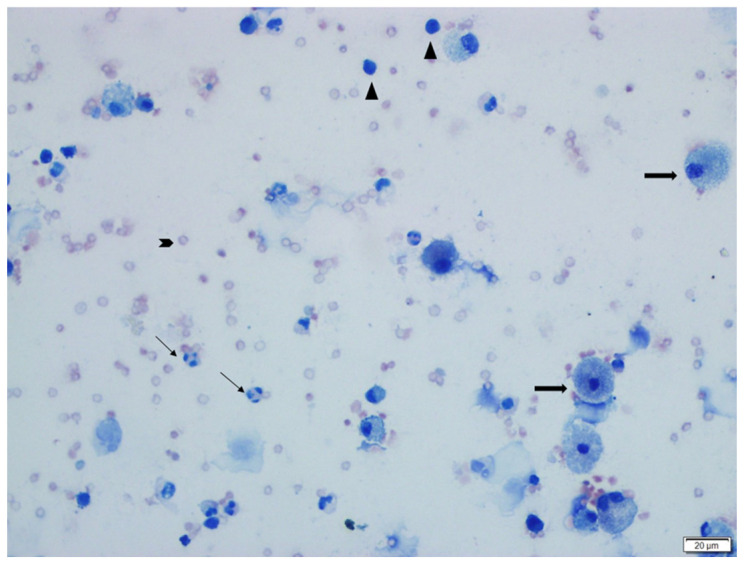
This image shows epithelial cells (thick long arrows), neutrophils (thin long arrows), lymphocytes (arrowheads), and erythrocytes (blunt arrows) in the BALF cytology of a rat from the BLM + DW group (Group 2). Giemsa stain.

**Figure 3:**
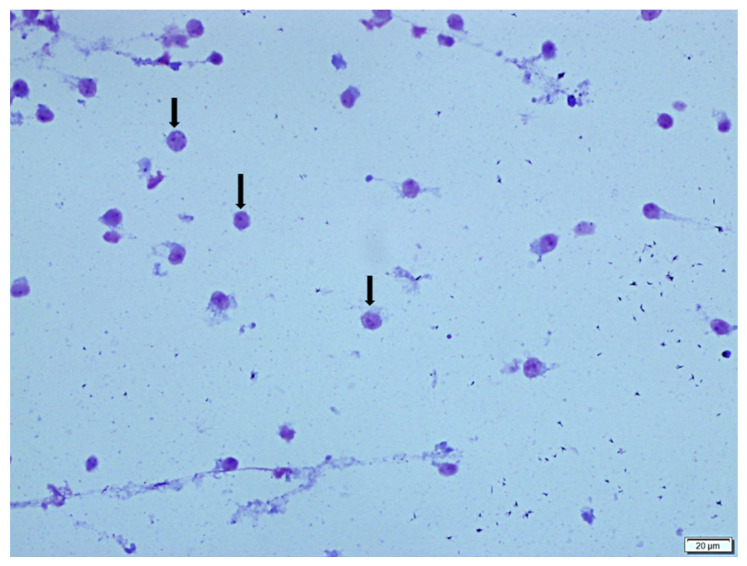
This image shows macrophages (long arrows) in the BALF cytology of a rat from the SS + DW group (Group 1). Giemsa stain.

**Figure 4:**
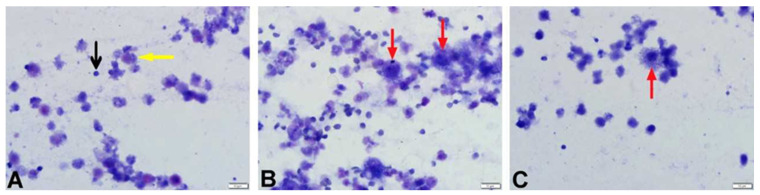
Microscopic appearance of BALF cytology from rats in the SS + DW, BLM + DW, and BLM + Tyrosol_80_ groups. (**A**). Cytology of a rat from the SF + DW group showing lymphocytes (black arrow) and macrophages (yellow arrow). (**B**). Foamy macrophages (red arrows) observed in the BALF cytology of a rat from the BLM + DW group. (**C**). A foamy macrophage (red arrow) observed in the BALF cytology of a rat from the BLM + Tyrosol80 group.

The cytological evaluations of BALF stained with Giemsa are presented in Table 2, showing the proportions of lymphocytes, macrophages, neutrophils, and epithelial cells within the total nucleated cell count (a minimum of 200 cells counted per slide). The BALF samples obtained from the control group were clear and light-colored, whereas in the BLM + DW group, the BALF appeared turbid, with some samples showing a hemorrhagic appearance. In this group, the turbid and hemorrhagic BALF samples were noted to have higher proportions of lymphocytes, neutrophils, and epithelial cells, whereas the proportion of macrophages was lower. Lymphocytes were small in size with round nuclei and scant cytoplasm; neutrophils had segmented nuclei and fine granules; macrophages were large with abundant cytoplasm; and epithelial cells were larger with prominent nuclei and ample cytoplasm. Foam macrophages, compared to other macrophages, had more extensive, pale cytoplasm filled with vacuoles. Compared to the control group (SS + DW), the BLM + DW group showed a statistically significant increase (*p* < 0.05) in the proportions of lymphocytes, neutrophils, and epithelial cells (Figure 2), along with a decrease in the macrophage ratio (Figure 3). When compared to the BLM + DW group, the groups treated with BLM + Tyrosol_20_, BLM + Tyrosol_40_, and BLM + Tyrosol_80_ exhibited a reduction in the percentages of these cells. Notably, the reductions observed in the BLM + Tyrosol_40_ and BLM + Tyrosol_80_ groups were statistically significant (*p* < 0.05). Moreover, most macrophages observed in the BLM + DW group were foam cells (Figure 4A,B), whereas their numbers decreased in the tyrosol-treated groups in a dose-dependent manner (Figure 4C). A clear relationship was observed between the cellular composition of BALF and pulmonary fibrosis. In groups BLM + DW, BLM + Tyrosol_20_, BLM + Tyrosol_40_, and BLM + Tyrosol_80_, elevated proportions of lymphocytes, neutrophils, and epithelial cells were identified in BALF cytology. Correspondingly, these groups exhibited higher histopathological scores, as evaluated using the scoring system described by Ashcroft et al. (1988) [17]. The scores were 5.61, 4.68, 4.27, and 3.03 for groups BLM + DW, BLM + Tyrosol_20_, BLM + Tyrosol_40_, and BLM + Tyrosol_80,_ respectively (unpublished data).

### 3.2. Biochemical Analysis

Statistical data regarding antioxidant (SOD, GPx, and CAT) and oxidative (MDA) parameters measured in BALF via the ELISA method are presented in Table 3. Compared to the control group (SS + DW), bleomycin administration significantly increased MDA levels in BALF (*p* < 0.05), while causing a reduction in the levels of SOD, GPx, and CAT. Among these, the decreases in SOD and CAT levels were statistically significant (*p* < 0.05). When compared to the BLM + DW group, the BLM + Tyrosol_20_, BLM + Tyrosol_40_, and BLM + Tyrosol_80_ groups showed statistically significant decreases in MDA levels and increases in SOD, GPx, and CAT levels. These increases were dose-dependent, with statistically significant differences particularly noted in the BLM + Tyrosol_40_ and BLM + Tyrosol_80_ groups (*p* < 0.05).

Statistical data for IL-1β, IL-6, IL-13, TNF-α, and TGF-β1 parameters in BALF, measured using ELISA, are presented in Table 4. Compared to the control group (SS + DW), bleomycin administration (BLM + DW group) led to an increase in all measured cytokines, with the increases in TGF-β1 and IL-6 levels reaching statistical significance (*p* < 0.05). When compared to the BLM + DW group, cytokine levels decreased dose-dependently in the BLM + Tyrosol_20_, BLM + Tyrosol_40_, and BLM + Tyrosol_80_ groups. Among these, only the reduction in IL-6 levels in the BLM + Tyrosol_80_ group was statistically significant (*p* < 0.05).

**Table 1:** Characteristics of primary antibodies used in ELISA analyses.

Antibody	Manufacturer Company	Analysis Range	Analysis Sensitivity	Wavelength	Catalog No.
**GPX**	Sunred	0.8 ng/mL→200 ng/mL	0.723 ng/mL	450 nm	201-11-1705
**SOD**	Sunred	0.5 ng/mL→100 ng/mL	0.415 ng/mL	450 nm	201-11-0169
**CAT**	Sunred	1 ng/mL–300 ng/mL	0.866 ng/mL	450 nm	201-11-5106
**MDA**	Sunred	0.3 nmol/mL→65 nmol/mL	0.208 nmol/m	450 nm	201-11-0157
**IL-1β**	Sunred	15 ng/L→3000 ng/L	10.135 ng/L	450 nm	201-11-0108
**IL-6**	Sunred	2 pg/mL–600 pg/mL	1.822 pg/mL	450 nm	201-11-0136
**IL-13**	Sunred	2 ng/L–360 ng/L	1.131 ng/L	450 nm	201-11-0113
**TNF α**	Sunred	8 ng/L→1000 ng/L	5.127 ng/L	450 nm	201-11-0765
**TGF β1**	Sunred	6 pg/mL→2000 pg/mL	5.126 pg/mL	450 nm	201-11-0779

**Table 2:** Statistical data of cell types observed in BALF cytology across experimental groups.

BALF CYTOLOGY									
		**Lymphocyte**		**Macrophage**		**Neutrophil**		**Epithelial Cell**	
**Groups**	**n**	$\overset{̿}{X}\pm S_{\overset{̿}{X}}$	MV.	$\overset{̿}{X}\pm S_{\overset{̿}{X}}$	MV.	$\overset{̿}{X}\pm S_{\overset{̿}{X}}$	MV.	$\overset{̿}{X}\pm S_{\overset{̿}{X}}$	MV.
**SS + DW**	9	5.142 ± 1.035	4.787 ^a^	92.331 ± 1.828	93.251 ^c^	0.307 ± 0.307	0.000 ^a^	2.154 ± 1.390	0.653 ^ab^
**BLM + DW**	8	36.673 ± 7.069	38.931 ^b^	38.016 ± 7.667	32.824 ^a^	19.412 ± 7.519	9.349 ^b^	5.898 ± 1.205	5.691 ^c^
**BLM + Tyrosol_20_**	8	30.521 ± 5.718	26.190 ^b^	49.369 ± 8.507	56.547 ^a^	16.403 ± 5.034	17.261 ^b^	3.706 ± 1.455	3.614 ^bc^
**BLM + Tyrosol_40_**	9	13.737 ± 3.162	12.962 ^a^	77.869 ± 3.405	77.847 ^b^	5.742 ± 0.782	5.076 ^a^	2.651 ± 0.337	3.151 ^ab^
**BLM + Tyrosol_80_**	9	13.997 ± 2.233	11.316 ^a^	80.802 ± 3.134	83.962 ^bc^	4.459 ± 1.639	2.690 ^a^	0.739 ± 0.330	0.238 ^a^
***P***		<0.001		<0.001		<0.001		<0.001	

$\overset{̿}{X}\pm S_{\overset{̿}{X}}$: Mean ± standard deviation; M.V: Median value. a, b, c: Differences between the values in the same column are statistically significant (*p* < 0.05).

**Table 3:** ELISA data of GPx, CAT, SOD, and MDA parameters in bronchoalveolar lavage fluid (BALF).

Oxidative Stress Parameters in Bronchoalveolar Lavage Fluid (BALF)									
		**GPX**		**SOD**		**CAT**		**MDA**	
**Groups**	**n**	$\overset{̿}{X}\pm S_{\overset{̿}{X}}$	MV.	$\overset{̿}{X}\pm S_{\overset{̿}{X}}$	MV.	$\overset{̿}{X}\pm S_{\overset{̿}{X}}$	MV.	$\overset{̿}{X}\pm S_{\overset{̿}{X}}$	MV.
**SS + DW**	9	49.499 ± 4.000	50.055	42.472 ± 2.538	43.505 ^c^	72.238 ± 2.247	71.915 ^b^	2.097 ± 0.037	2.097 ^a^
**BLM + DW**	8	41.593 ± 2.091	41.117	34.771 ± 0.396	34.723 ^a^	48.193 ± 2.797	48.658 ^a^	3.023 ± 0.141	3.074 ^c^
**BLM + Tyrosol_20_**	8	41.683 ± 2.468	42.517	34.733 ± 0.729	34.819 ^a^	49.992 ± 2.466	50.559 ^a^	2.684 ± 0.104	2.765 ^bc^
**BLM + Tyrosol_40_**	9	47.016 ± 2.685	46.867	37.230 ± 0.636	37.727 ^ab^	63.037 ± 0.9231	62.572 ^b^	2.404 ± 0.123	2.422 ^ab^
**BLM + Tyrosol_80_**	9	47.672 ± 1.582	47.254	40.826 ± 1.040	40.998 ^bc^	70.803 ± 1.783	71.025 ^b^	2.292 ± 0.205	2.209 ^ab^
***P***		0.305		0.008		0.005		0.025	

$\overset{̿}{X}\pm S_{\overset{̿}{X}}$: Mean ± standard deviation; MV: Median value. a, b, c: Differences between the values in the same column are statistically significant (*p* < 0.05).

**Table 4:** ELISA data of IL-1β, IL-6, IL-13, TNF-α, and TGF-β1 parameters in bronchoalveolar lavage fluid (BALF).

Parameters in Bronchoalveolar Lavage Fluid (BALF)											
		**TNF-α**		**TGF-β1**		**IL-1β**		**IL-6**		**IL-13**	
**Groups**	**n**	$\overset{̿}{X}\pm S_{\overset{̿}{X}}$	MV.	$\overset{̿}{X}\pm S_{\overset{̿}{X}}$	MV.	$\overset{̿}{X}\pm S_{\overset{̿}{X}}$	MV.	$\overset{̿}{X}\pm S_{\overset{̿}{X}}$	MV.	$\overset{̿}{X}\pm S_{\overset{̿}{X}}$	MV.
**SS + DW**	9	243.515 ± 15.186	246.685	581.237 ± 25.448	561.822 ^a^	532.503 ± 45.884	575.471	121.809 ± 4.743	119.844 ^a^	78.972 ± 3.171	80.793
**BLM + DW**	8	280.220 ± 11.861	275.881	692.406 ± 13.338	677.736 ^c^	691.662 ± 64.706	621.509	133.402 ± 2.976	134.204 ^b^	83.881 ± 4.566	90.118
**BLM + Tyrosol_20_**	8	278.289 ± 26.475	260.693	686.719 ± 21.722	694.398 ^bc^	698.239 ± 52.770	690.566	128.266 ± 4.624	133.772 ^ab^	84.019 ± 4.041	81.890
**BLM + Tyrosol_40_**	9	272.805 ± 17.416	262.905	685.498 ± 24.981	700.919 ^bc^	619.974 ± 44.436	598.490	127.202 ± 1.235	128.482 ^ab^	82.198 ± 4.967	82.439
**BLM + Tyrosol_80_**	9	256.653 ± 18.227	280.009	648.214 ± 27.971	643.686 ^abc^	544.780 ± 40.739	560.125	120.168 ± 2.096	120.546 ^a^	81.912 ± 4.582	79.916
***P***		0.504		0.008		0.182		0.073		0.334	

$\overset{̿}{X}\pm S_{\overset{̿}{X}}$: Mean ± standard deviation; MV: Median value. a, b, c: Differences between the values in the same column are statistically significant (*p* < 0.05).

## 4. Discussion

In this study, bleomycin administration caused a statistically significant increase in the proportions of lymphocytes, neutrophils, and epithelial cells in BALF, with lymphocytes being the predominant cell type in cytological evaluation. Our observations indicate that groups with high lymphocyte rates in the BALF (BLM + DW and BLM + Tyrosol_20_) also show signs of pulmonary fibrosis (unpublished data). These findings suggest a strong association between increased inflammatory cell ratios in BALF and the severity of lung tissue damage. In the tyrosol-treated groups, tyrosol reduced the proportions of lymphocytes, neutrophils, and epithelial cells in a dose-dependent manner, while increasing the proportion of macrophages. As previously reported by Giri et al. (1986), macrophages were the predominant cells in BALF from control animals, whereas polymorphonuclear cells (PMNs) became dominant following bleomycin exposure [18]. In our study, although the BLM group showed an increase in PMNs, lymphocytes remained the predominant cell type. It is known that neutrophil levels typically rise in BALF in fibrotic lung diseases, and in some cases, lymphocytes and eosinophils also increase [7,19]. Some researchers suggest that a dominance of PMNs in BALF may indicate bacterial pneumonia [20]. Regarding macrophage proportions, a reduction was observed in the BLM group compared to the control, and the macrophages present were predominantly foamy. Tyrosol administration reduced the number of foamy macrophages in a dose-dependent manner. The presence of foamy macrophages in BALF has been reported as an early marker of bleomycin-induced lung injury and is associated with pathological processes such as fibroblast activation and the release of profibrotic cytokines like TGF-β and IL-6 [21,22,23]. Romero et al. (2015) also indicated that foamy macrophages may play a role in the pathogenesis of pulmonary fibrosis and that therapies targeting these cells may help limit disease onset or progression. These macrophages are thought to emerge in response to alveolar epithelial damage and contribute to the inflammatory phase of lung injury [21]. Some researchers have reported significant increases in IL-1β and IL-6 levels [24], while others have documented elevations in IL-13, TNF-α, and TGF-β levels [25,26,27]. In our study, although an increase was observed in all cytokines, only the elevations in IL-6 and TGF-β were found to be statistically significant. These discrepancies may be attributed to biological response variability arising from differences in the dose of bleomycin used in experimental models, the types of ELISA kits employed, the timing of cytokine measurements, and variations in analytical techniques. Yong-Kim et al. (2017) reported that tyrosol significantly reduced the levels of TNF-α, IL-6, and IL-1β in BALF and lung tissue in a lipopolysaccharide (LPS)-induced acute lung injury model [28]. Similarly, Gabbia (2024) and Kutlu et al. (2021) demonstrated that tyrosol administration reduced TNF-α, IL-6, and TGF-β1 levels in models of chronic liver injury [29,30]. In the study, all cytokine levels assessed in BALF samples from tyrosol-treated groups showed a decreasing trend; however, this decrease reached statistical significance only for IL-6. This selective effect may be related to the fact that IL-6 is a proinflammatory cytokine that plays a key role in the early phase of inflammation [31], and it may exhibit a faster and more prominent response to anti-inflammatory agents. Previous studies have suggested that tyrosol reduces IL-6 expression by inhibiting NF-κB and MAPK signaling pathways [10]. Although these pathways were not directly measured in our study, the significant reduction in IL-6 may be associated with these mechanisms. Decreases were also observed in TNF-α, TGF-β1, IL-1β, and IL-13 levels; however, these changes did not reach statistical significance. These findings suggest that tyrosol may exert a weaker effect on these cytokines compared to IL-6. Alternatively, technical and methodological factors such as the sensitivity limits of the ELISA kits, sample size, or biological variability may also have contributed to these outcomes. It has been widely reported that bleomycin exposure disrupts redox balance by reducing the antioxidant defense system, leading to increased hydroxyl radical production, lipid peroxidation, and cellular damage [32,33]. Consistent with this, the present study found increased BALF MDA levels and decreased GPx, CAT, and SOD levels in the BLM group compared to controls. Similarly to previous studies, our findings indicate that BLM significantly impairs antioxidant enzyme activity in BALF. Some researchers [34,35] have reported decreases in SOD and CAT levels, while others [36] have also observed reduced GPx levels. Limited studies on the effects of tyrosol on the oxidant/antioxidant system have indicated that it may exert a protective effect on the liver and kidneys by reducing oxidative stress or enhancing antioxidant defenses [37,38,39,40]. In our study, compared to the BLM-only group (Group 2), the tyrosol-treated groups (Groups 3, 4, and 5) showed significant reductions in MDA levels and increased activity of GPx, SOD, and CAT enzymes. These findings demonstrate that tyrosol reduces oxidative stress and enhances antioxidant activity in BALF, with effects varying according to dosage.

In this study, tyrosol at a dose of 80 mg/kg was found to exhibit a more pronounced protective effect on BALF composition compared to other doses. However, considering that the toxic dose threshold of tyrosol is relatively high [41], it is suggested that its efficacy at higher doses should also be investigated. Moreover, the study provides preliminary evidence indicating that tyrosol may exert anti-inflammatory effects at the alveolar level and could be considered a potential protective agent in early-stage lung injury.

## 5. Conclusions

In conclusion, intratracheal administration of 4 mg/kg bleomycin in Sprague Dawley rats increased oxidative stress and proinflammatory cytokine levels in BALF. Tyrosol, in a dose-dependent manner, reduced MDA levels while increasing GPx, SOD, and CAT levels, indicating its antioxidant potential. Moreover, tyrosol led to a significant reduction in IL-6 levels and a limited reduction in IL-1β, IL-13, TGF-β1, and TNF-α levels, supporting its anti-inflammatory effects.

## Data Availability

The data presented in this study are available upon request from the corresponding author (E.E).
